# BYPASS VS. SLEEVE AND ITS EFFECTS IN NON-ALCOHOLIC FATTY LIVER DISEASE: WHAT IS THE BEST TECHNIQUE?

**DOI:** 10.1590/0102-672020200003e1549

**Published:** 2021-01-15

**Authors:** Marcos Bertozzi GOLDONI, Paulo Roberto Ott FONTES, Marcela Menuci GUIMARÃES, João Alfredo DIEDRICH-NETO, Tiele NOGUEIRA, Uirá Fernandes TEIXEIRA, Caroline Becker GIACOMAZZI, Guillermo KISS, Sérgio Ricardo PIONER, Pablo Duarte RODRIGUES

**Affiliations:** 1 Federal University of Health Sciences of Porto Alegre, Porto Alegre, RS, Brazil; 2 Irmandade Santa Casa de Misericórdia de Porto Alegre, Porto Alegre, RS, Brazil

**Keywords:** Bypass, Sleeve, Non-alcoholic fatty liver disease, Morbid obesity, Bariatric surgery, Bypass, Sleeve, Doença hepática gordurosa não alcoólica, Obesidade mórbida, Cirurgia bariátrica

## Abstract

**Background:**

Strongly associated with obesity, non-alcoholic fatty liver disease is considered the hepatic manifestation of the metabolic syndrome. It presents as simple steatosis and steatohepatitis, which can progress to cirrhosis and its complications. Among the therapeutic alternatives is bariatric surgery.

**Aim:**

To compare the effect of the two most frequent bariatric procedures (sleeve and bypass) on liver disease regarding to epidemiological, demographic, clinical and laboratory parameters.

**Methods:**

The results of intraoperative and 12 months after surgery liver biopsies were used. The NAFLD activity score (NAS) was used to assess and compare the stages of liver disease.

**Results:**

Sixteen (66.7%) patients underwent Bypass procedure and eight (33.3%) Sleeve. It was observed that the variation in the NAFLD activity score was significantly greater in the Bypass group than in Sleeve (p=0.028) and there was a trend regarding the variation in fibrosis (p=0.054).

**Conclusion:**

Both surgical techniques were effective in improving the hepatic histology of most operated patients. When comparing sleeve and bypass groups, bypass showed better results, according to the NAS score.

## RESUMO

**Racional:**

Fortemente associada à obesidade, a doença hepática gordura não alcoólica é considerada a manifestação hepática da síndrome metabólica. Ela apresenta-se como esteatose simples e esteato-hepatite, podendo evoluir para cirrose e suas complicações. Entre as alternativas terapêuticas está a cirurgia bariátrica.

**Objetivo:**

Comparar o efeito sobre a doença hepática dos dois procedimentos bariátricos mais frequentes - sleeve e bypass *-* e comparar dados epidemiológicos, demográficos, parâmetros clínicos e laboratoriais.

**Métodos:**

Utilizou-se o resultado das biópsias hepáticas realizadas no intra-operatório e 12 meses após a operação. O NAFLD activity score foi utilizado para avaliar e comparar os estágios da doença hepática.

**Resultados:**

Dezesseis (66,7%) pacientes foram submetidos ao bypass e oito (33,3%) ao sleeve. Observou-se melhora significativa no IMC e glicemia nas duas técnicas cirúrgicas enquanto que os níveis de fosfatase alcalina, ferritina, Gama-GT e TGP reduziram com significância apenas no grupo bypass*.* A redução no NAFLD activity score foi significativamente maior no grupo bypass que no sleeve (p=0,040).

**Conclusão:**

Ambas as técnicas foram eficazes em promover a melhora da histologia hepática da maior parte dos pacientes operados. Quando comparadas o bypass apresentou melhores resultados.

## INTRODUCTION

Non-alcoholic fatty liver disease (NAFLD) has a global distribution and is considered nowadays the most common liver disease in the west industrialized countries, probably because of the association with obesity, type 2 diabetes (DM2), dyslipidemia and metabolic syndrome. The prevalence of NAFLD is reported as 6-35%, with an average of 20%, in the general population^33^.

There is a strong evidence that NAFLD represents the hepatic component of the metabolic syndrome, characterized by obesity, hyperinsulinemia, insulin resistance (IR), DM2, hypertriglyceridemia and systemic arterial hypertension (SAH). Obesity is a common and well-documented risk factor for NAFLD. In patients with severe obesity undergoing bariatric surgery, the prevalence of NAFLD can exceed 90%, with up to 50% of patients presenting steatohepatitis (NASH) and 5% cirrhosis^6^ ^,^ ^25^.

Abdominal ultrasound is widely used as the first line in the investigation of NAFLD and evidence the accumulation of fat when more than 33% of hepatocytes have steatosis. Liver biopsy is the gold standard method for the diagnosis of NAFLD, being able to assess the degree of fatty infiltration, hepatocellular damage, inflammation and fibrosis. The presence of hepatocellular ballooning in association with steatosis is the key to the differential diagnosis between simple steatosis and NASH^29^.

All patients with NAFLD should undergo interventions to promote a healthier lifestyle and strict control of the metabolic risk factors associated with NAFLD^17^. Weight loss is the most important factor. Relatively small losses of approximately 7-10% of body weight in 12 months seem to be effective in improving NAFLD already^11^ ^,^ ^20^.

Obesity and DM2, isolated or linked by metabolic syndrome, are the most important risk factors in the genesis of NAFLD. The role of bariatric surgery comes up in this context. Several consistent studies over the past few years, culminating in the publication of Philip Schauer in 2017^31^, have shown that surgical treatment is more effective in weight loss and in the control of glycemia and metabolic syndrome than intensive and optimized clinical treatment in the short, medium and long terms.

Although there are still no randomized clinical trials evaluating the role of bariatric surgery in the treatment of NAFLD, there are several retrospective and prospective studies in the literature, in addition to a few meta-analyzes, which evaluate the effect of surgical treatment on NAFLD^1^ ^-^ ^3^ ^,^ ^5^ ^,^ ^8^ ^-^ ^10^ ^,^ ^12^ ^-^ ^14^ ^,^ ^16^ ^,^ ^20^ ^,^ ^21^ ^,^ ^23^ ^-^ ^26^ ^,^ ^32^ ^,^ ^34^. The AASLD and EASL guidelines define bariatric surgery as a therapeutic option in NAFLD, mainly in those patients who did not respond to conservative clinical treatment^11^ ^,^ ^20^.

Roux-en-Y bypass (bypass) and vertical gastrectomy (sleeve) have been compared in several studies to determine which one has the greatest benefit in weight loss or in resolving obesity-related comorbidities. However, there is few knowledge about which of these techniques is associated with the best results on NAFLD. There are few comparative studies and these are small series with a large number of biases, such as heterogeneity of groups and assessment systems^7^ ^,^ ^15^ ^,^ ^28^.

In a group of obese patients, the main objective of the present study was to compare the bariatric sleeve and Roux-en-Y bypass techniques and their effects on NAFLD, also in epidemiological, demographic, clinical and laboratory parameters.

## METHODS

The study was submitted to and approved by the Ethics Committee of the institution, where it was carried out. CAAE: 90475215500005335.

### Patients selection

A retrospective study that evaluated patients undergoing bariatric surgical procedures by the same surgical team, between 2014 and 2016, at Irmandade da Santa Casa de Misericórdia de Porto Alegre, Porto Alegre, RS, Brazil.

Only patients with intraoperative and postoperative liver biopsy were included. The average time between biopsies was 12 months.

Demographic characteristics (gender and age), clinical parameters (weight, height, BMI, SAH, weight loss, excess weight lost) and laboratory (platelets, ALP, ferritin, GGT, AST, ALT), histopathological analysis and NAS score^16^ were collected and analyzed.

### Operative technique

All procedures were performed laparoscopically.

The sleeve surgery was made with the dissection of the greater gastric curvature 3 cm from the pylorus to the esophagogastric flexure. The gastric transection was calibrated with a 36F bougie and reinforcing the staple line with continuous suture with Caprofyl^®^ 2.0.

In the bypass surgery, a 4 cm gastric pouch was done using a 36F bougie. The Roux limb used to be 100cm and the biliopancreatic limb used to be 150 cm. Gastroenteric anastomosis was performed laterally with a linear stapler calibrated by a 36F bougie.

### Histopathological evaluation

All liver samples were analyzed in the hospital’s pathology laboratory by a single professional experienced in liver pathology. The NAFLD activity score (NAS) was used to assess and compare the stages of liver disease^18^. It proposes the characterization of NAFLD regarding the degree of steatosis, the presence of ballooning and inflammation activity. NAS scores the histological analysis from 0 to 3: degree of steatosis (0-3), lobular inflammation (0-3) and ballooning (0-2). The degree of fibrosis was assessed semi-quantitatively with a scale of 0 to 4.

### Statistical analysis

Categorical results were presented using frequency and percentage and were analyzed using the X^2^ test (chi-square). The likelihood-ratio test was performed in comparisons when there were more than 20% of the cells with an expected value below 20%. Quantitative results were displayed using means±standard deviations and when data followed normal distribution it was analyzed using Student’s t tests for paired and independent samples; when non-parametric, the Wilcoxon and Mann-Whitney tests were used. The normality of the data was verified using the Shapiro-Wilk test. The variation in the NAS score was calculated using the difference between the postoperative and the intraoperative score. The analyzes were performed using SPSS software, version 21 and significant results were considered when p <0.05.

## RESULTS

Twenty-four obese patients who underwent bariatric surgery were evaluated, of which 16 (66.7%) undergoing bypass and eight sleeve, with a mean segment time of 21.3±16 months and 15.5±12.5 months, respectively (p=0.380). 68.8% of patients undergoing bypass were women, on the sleeve group, 75% (p=0.571); 31.3% were hypertensive vs. 37.5% in the sleeve (p=0.553); in the bypass group, age and maximum weight were 38.6±11.3 years and 119.1±14.1 kg respectively vs. 36.7±8.4 years, p=0.692 and 119.1±13.5, p=0.999 in the sleeve group. Furthermore, the two techniques were also similar in terms of weight loss (44.2±15.1 kg vs. 37.9±7.4 kg, p=0.180, respectively) and the excess weight lost (84.4±29, 8 kg vs. 83.2±26.8 kg, p=0.904, respectively).

Table 1 shows the results of the clinical and laboratory parameters in the preoperative period and after the surgical procedures. In both periods and in all parameters analyzed, no difference was found between the techniques with statistical significance. However, there were significant reductions in BMI and glycemia in the two techniques analyzed, while reductions in alkaline phosphatase, ferritin, GGT and ALT were significant only in the bypass group.

**TABLE 1:** Pre and postoperative clinical and laboratory parameters according to the type of operation performed.

Parameter	Technique	Pre-op	Post-op	p
BMI (kg/m²)	Bypass	44.3±4.2	27.9±4.1	<0.001
	Sleeve	42.1±4.1	28.6±5.8	<0.001
	p	0.239	0.730	
Platelets (x1000)	Bypass	270±59	239±66	0.170
	Sleeve	283±45	279±71	0.832
	p	0.606	0.191	
ALP (U/L)	Bypass	92.2±30.7	72.8±16.8	0.010
	Sleeve	105.5±20.6	103.0±70.3	0.910
	p	0.283	0.268	
Ferritin (ng/ml)	Bypass	227.1±150.3	121.1±86.4	0.011
	Sleeve	232.6±76.1	133.6±103.1	0.069
	p	0.854	0.976	
GGT (U/L)	Bypass	55.9±31.1	19.1±10.8	0.000
	Sleeve	111.0±151.8	64.5±116.3	0.123
	p	0.312	0.197	
Glycemia (mg/dl)	Bypass	99.4±17.2	82.1±9.8	0.001
	Sleeve	102.75±10.2	90.2±8.6	0.007
	p	0.616	0.059	
AST (U/L)	Bypass	30.8±16.2	25.5±6.9	0.338
	Sleeve	33.5±12.2	39.0±27.9	1.000
	p	0.408	0.358	
ALT (U/L)	Bypass	42.1±20.6	28.3±10.6	0.023
	Sleeve	39.9±12.5	31.6±13.2	0.207
	p-	0,806	0,602	

BMI=body mass index; ALP=alkaline phosphatase; GGT=gamma glutamyl transpeptidase; AST=aspartate aminotransferase; ALT=alanine aminotransferase.

**FIGURE 1:**
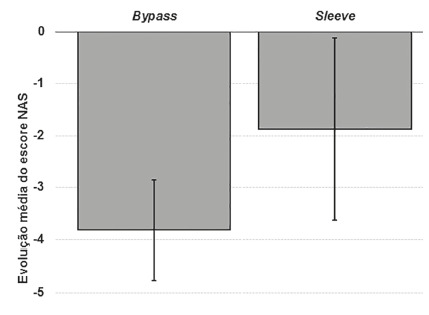
Evolution of NAFLD degree

Evaluating the evolution of the NAFLD degree on the second biopsy compared to the first, 19 (79.2%) patients showed improvement, two (8.3%) remained in the same degree and three (12.5%) worsened. There was a significant association between the evolution of NAFLD and the surgical procedure performed (p=0.024). In the sleeve group, three (37.5%) worsened the level of NAFLD vs. no patient in the bypass group (Figure 1).

Table 2 shows the differences in the NAS^16^ classification between the first and second biopsies. In all categories, a significant reduction was observed when the technique was bypass. However, in the sleeve group, only steatosis and the NAS score showed significant reductions.

**TABLE 2:** Comparison between the first and second liver biopsies

NAS classification	First	Second	Change	p
Bypass				
Steatosis	2.0 ± 0.8	0.2 ± 0.4	-1.81 ± 0.91	<0.001
Inflammation	1.2 ± 0.7	0.5 ± 0.5	-0.75 ± 0.77	0.005
Ballooning	1.5 ± 0.9	0.2 ± 0.7	-1.25 ± 1.00	0.002
Fibrosis	0.9 ± 1.0	0.2 ± 0.7	-0.69 ± 0.87	0.015
NAS score	4.8 ± 1.7	0.9 ± 1.3	-3.81 ± 1.80	<0.001
Sleeve				
Steatosis	1.5 ±0.7	0.2 ± 0.5	-1.25 ± 0.89	0.014
Inflammation	0.7 ± 0.9	0.7 ± 0.5	-0.13 ± 0.64	0.564
Ballooning	1.0 ± 1.1	0.5 ± 0.9	-0.50 ± 1.41	0.317
Fibrosis	0.4 ± 0.7	0.6 ± 0.9	0.25 ± 1.39	0.581
NAS score	3.2 ± 1.8	1.4 ± 1.7	-1.87 ± 2.10	0.048

**FIGURE 2:**
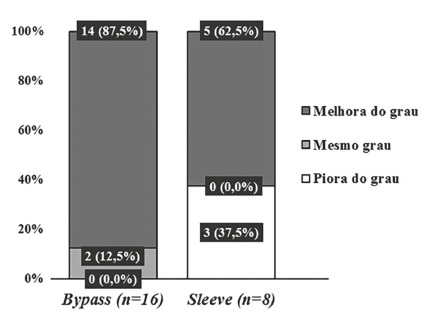
Comparison of the mean variation of the NAS score

The intensity of variations was compared between groups with no significant difference between them (p>0.05), except for the NAS score shown in Figure 2, whose reduction observed in the bypass was significantly higher than in the sleeve (-3,81±1,80 vs. -1,87±2,10, respectively; p=0,040).

## DISCUSSION

In our country, bypass is still the most performed bariatric technique; but, in recent years, there has been an important increase in the number of vertical gastrectomy, which follows the worldwide trend of technical preference nowadays^4^. 

Historically, patients with morbid obesity or those with clinical signs and symptoms of metabolic syndrome had in the operations of mixed character (bypass) - with restrictive and disabsorptive component -, their most frequent indication. Purely restrictive procedure - vertical gastrectomy - a relatively newer technique, was initially indicated for patients with grade II obesity or very high BMI levels greater than 60 kg/m², those in which the necessary weight loss was not too much or those that mixed operations has became impractical due to the great excess of weight. Thus, sometimes it becomes difficult to compare the results between the techniques, since the patients generally presented important clinical and epidemiological differences, making statistically different groups.

In the present study, evaluating the demographic characteristics of both groups - bypass and sleeve - it was noticed that there were no statistical differences between them. Gender, breed and age were similar. SAH was present in approximately 30% of patients. There were no diabetic patients and the mean blood glucose was practically the same in both groups, 100 mg/dl. Regarding weight, BMI, laboratory tests - platelets, alkaline phosphatase, ferritin, gamma-GT, glycemia, AST and ALT preoperatively - the groups also showed similarity. This parity between the groups contributes to a more reliable comparative analysis with a lower incidence of bias.

Already widely discussed and consolidated in the literature, these bariatric procedures have an important effect on weight loss. In the present study, in the mean follow-up of 12 months, the BMI showed an average reduction of 16.3±5.23 kg/m^2^ and 13.4±2.7 kg/m^2^ in the bypass and sleeve, respectively. This reduction represents the average loss of approximately 84% of excess body weight for both techniques. As previously published by several authors, and more recently by Peterli et al^27^, the tendency is not to have statistical differences between bypass and sleeve considering weight loss in the short, medium and long terms.

Analyzing blood glucose separately, it was observed that both surgical techniques were effective, statistically reducing serum glucose levels by 17.3±15.7 mg/dl and 12.5±9.4 mg/dl, considering bypass and sleeve respectively. When comparing the final results between the techniques, there is a favorable trend towards bypass (p=0.059), probably depending on a larger sample. In recent years, it has been associated with techniques with intestinal deviation, a condition of metabolic change that would provide better results on glycemic control, compared to purely restrictive techniques.The increase in intestinal hormones such as glucagon-like peptide-1 (GLP-1) and *peptide*YY(PYY), the result of the food’s earlier contact with the ileum, would be responsible for this fact. However, more recently, although there is no established pathophysiological explanation, there are studies showing a similar result between the sleeve and bypass regarding glycemic control^30^.

Analyzing the other laboratory tests evaluated in the study, we observed that in the bypass group there was a significant improvement in the levels of alkaline phosphatase, ferritin, gamma-GT and ALT, which did not occur in the sleeve. AST and platelets showed no significant changes.

As previously mentioned, the mechanism by which bariatric surgery performs potential treatment for NAFLD is complex and has not been fully elucidated yet. It is probably related to improvement of factors such as insulin resistance, lipid profile, inflammation, weight loss and adipokines, which are directly involved in the development of metabolic syndrome and, consequently, in its hepatic phenotype, NAFLD. These changes are seen right after the surgical procedure, during the phase when weight loss is not yet significant. It is well established that weight loss plays a fundamental role in the control of metabolic abnormalities; however, endocrinophysiological changes resulting from the operation may have a more relevant effect in the long term on NAFLD. These findings have been confirmed by several studies published in the international literature. As in the present study, NAFLD in its several stages, simple steatosis, NASH and fibrosis, showed significant improvement after the bariatric procedure, be it bypass or sleeve ^1^ ^,^ ^9^ ^,^ ^10^ ^,^ ^13^ ^,^ ^14^ ^,^ ^16^ ^,^ ^19^ ^,^ ^21^ ^,^ ^22^ ^,^ ^23^.

However, it can be noticed that part of the operated patients does not improve or could get a worse level of NAFLD, which ranges from 7-20% of patients^22^ ^,^ ^23^ ^,^ ^32^. In the present study, it was observed that five patients (20.8%) did not demonstrate complete resolution of NAFLD. Of these, three (12.5%) showed disease progression, all in the sleeve group.

In the quantitative analysis of NAFLD, the bypass group showed between the first and second biopsies, significant improvement in all NAS parameters (steatosis, inflammation and ballooning), in fibrosis and in the NAS score itself. In the sleeve group, statistical significance was observed only in the steatosis and in the NAS score. Comparing the change in the NAS score between the first and second biopsies, it was observed that this was significantly higher in the bypass group (-3.81±1.80 vs. -1.87±2.10; p=0.040). 

In line with the literature, in the present study there was a significant improvement in NAFLD after bariatric procedures, both in the purely restrictive operation (sleeve) and in the mixed operation (bypass).

However, unlike the previous findings published by Froylich^15^, Billeter^7^ and Praveen^28^, who presented the operative results on NAFLD comparing the sleeve and bypass techniques and did not find statistical differences or tended to the sleeve as the best results procedure, in the present study, the bypass had better effects on NAFLD, including a statistical difference when considering the evolution of the NAS score. There is currently no clear explanation, but it is likely that anatomical changes, particularly duodenal exclusion, increased flow of nutrients to the distal small intestine and the resulting hormonal and metabolic changes related to bypass are responsible for these findings.

The retrospective nature of the study, the small number of patients in the groups and the possible selection biases are important limitations that must be considered when analyzing these results.

## CONCLUSION

Sleeve and bypass were effective in restoring the liver histology of most operated patients. BMI and blood glucose showed significant improvement in both surgical techniques, with no significant difference in results between them. The levels of alkaline phosphatase, ferritin, gamma-GT and ALT decreased significantly only in the bypass group, but they did not differ from the values​​observed in the sleeve. However, the bypass showed significantly better results in the change of the NAS score than the sleeve.
